# A Middle East systematic review and meta-analysis of prevalence and antibiotic susceptibility pattern in MRSA *Staphylococcus aureus* isolated from patients with cystic fibrosis

**DOI:** 10.1186/s41043-022-00305-x

**Published:** 2022-06-28

**Authors:** Yousef Nikmanesh, Afsaneh Foolady Azarnaminy, Pourya Avishan, Mohammadreza Taheri, Paniz Sabeghi, Ehsan Najibzadeh, Azad Khaledi

**Affiliations:** 1 Gastroenterohepatology Research Center, Shiraz University of Medical Sciences, Shiraz, Iran; 2 Department of Anesthesiology, Social Security Organization Hospital, Ardabil, Iran; 3 Faculty of Medicine, Tabriz University of Medical Sciences, Tabriz, Iran; 4 School of Medicine, Zahedan University of Medical Sciences, Zahedan, Iran; 5 Shiraz University of Medical Sciences, Shiraz, Iran; 6 Medical School, Tehran University of Medical Sciences, Tehran, Iran; 7 Infectious Diseases Research Center, Kashan University of Medical Sciences, Kashan, Iran; 8 Department of Microbiology and Immunology, School of Medicine, Kashan University of Medical Sciences, Kashan, Iran

**Keywords:** Cystic fibrosis, Patient, Middle East, *Staphylococcus aureus*, Antibiotic resistance

## Abstract

**Background:**

This study aimed to determine the prevalence and antibiotic resistance patterns in *Staphylococcus aureus* isolated from patients with cystic fibrosis in Middle Eastern countries.

**Methods:**

A systematic search was conducted in the PubMed, Web of Science (ISI), and Scopus databases for studies presenting the prevalence of MRSA strains, antibiotic resistance pattern in *S. aureus* strains isolated from patients who suffered from cystic fibrosis in Middle Eastern countries from 1999 to 10 June 2020. The following terms were used; prevalence, antibiotic resistance, antimicrobial drug resistance, drug resistance,* Staphylococcus aureus*, *S. aureus*, *Methicillin-resistant Staphylococcus aureus,* MRSA, cystic fibrosis, CF, and the Middle East. The meta-analysis was performed using Comprehensive Meta-analysis software (Version 3.3.070).

**Results:**

Patients’ age ranged from 1.6 to 18 years. Females were more than males. The prevalence of *S. aureus* was varied between 5.6 and 77.8%. The prevalence of *S. aureus* was varied between 5.6 and 77.8% in different countries. The combined prevalence of *S. aureus* in Middle East countries from 1999 to 2020 was reported by 40.9% (95% CI 29.6–53.1). The pooled prevalence of MRSA was reported at 18.6% (95% CI 1.1–82.6), *Z* = 0.9, *I*^2^ = 98.6, *Q* = 146.7. The highest combined resistance in *S. aureus* strains was reported to Penicillin G (94%), followed by Ciprofloxacin (54.9%).

**Conclusion:**

Regarding a quite prevalence of *S. aureus* and an intermediate prevalence of MRSA in CF patients, preventive measures and health policies should be implemented in the Middle East area to prevent the spread of infections caused by MRSA strains in CF patients.

**Supplementary Information:**

The online version contains supplementary material available at 10.1186/s41043-022-00305-x.

## Background

Cystic fibrosis (CF) is an inherited multisystem dysfunction determined by abnormalities in exocrine gland function. A mutation in the cystic fibrosis conductance regulator (CFTR) gene sited on chromosome 7 in humans is the cause of cystic fibrosis (CF) [1]. Chronic sinopulmonary disease, pancreatic exocrine impairment, elevated sweat chloride, and male infertility are common disorders that resulted from CF [2]. Cystic fibrosis is the most frequent, lethal, genetic mutation which affects more than 70,000 people globally [3, 4]. In recent decades, advancements in disease management and new therapeutic developments have led to improved patients’ survival, with surviving into the late thirties, as around 50% of all persons with CF are now 18 years of age or older [2].

Significant advances have been made in the lives of people with CF over the past six decades, which was once a life-threatening disease of infants and young children. However, life expectancy for people with CF has gradually increased, However, the disease still affects the survival and quality of people’s lives and imposes a heavy health burden on patients and their families [5]. According to reports, most patients with CF acquire pathogens from the environment, especially medical settings when they stay in these centers for a long time [1].

*Staphylococcus aureus* and *Pseudomonas aeruginosa*, *Achromobacter xylosoxidans*, *Burkholderia cepacia* complex, and *Stenotrophomonas maltophilia* are the most common isolates retrieved from CF patients [6]. *S. aureus* is one of the first respiratory colonizers in people with CF [7]. This microorganism causes opportunistic infections in people with underlying diseases such as CF [8, 9]. MRSA; a type of bacteria that causes an infection that does not respond to common antibiotics, including methicillin, amoxicillin, and penicillin as opposed to methicillin-susceptible *Staphylococcus aureus* (MSSA) [10]. The findings suggest that not only it is difficult to treat infections caused by MRSA strains, but it is also now known that MRSA infection may exacerbate lung function [11, 12].

The Middle East region is a vast area with several countries of different ethnicities, races, cultures, and climatic diversity [13]. The local prevalence in countries in the Middle East area is changing, due to the outline of new strains with the intercontinental exchange of several clones [14]. Several studies from this region reported a prevalence rate between 10 and 35% [15–17]. MRSA is endemic in this district, and this causes an increase in the risk of domestic and global transmission [14].

Therefore, due to the widespread prevalence of *S. aureus* in cystic fibrosis patients, the determination of the prevalence of this bacterium and its antibiotic resistance pattern is of special importance. So, this study aimed to determine the prevalence of MRSA strains, antibiotic resistance patterns in *S. aureus* strains isolated from patients with cystic fibrosis in Middle Eastern countries.

## Material and methods

### Literature search

A systematic search was conducted in PubMed, Web of Science (ISI), and Scopus databases for studies presenting the prevalence of MRSA strains, antibiotic resistance pattern in *S. aureus* strains isolated from patients with CF in some Middle Eastern countries from 1999 to 10 June 2020. The following search terms were used: prevalence, antibiotic resistance, antimicrobial drug resistance, drug resistance*, Staphylococcus aureus*, *S. aureus*, *Methicillin-resistant Staphylococcus aureus,* MRSA, cystic fibrosis, CF, and the Middle East, Iran, Palestine, Kuwait, Qatar, Emirate United Arab, Saudi Arabia, Lebanon, Turkey, Yemen, Oman, Bahrain, Egypt, Iraq, Jordan, Syria, and Cyprus.

Search strategy in PubMed was as follows; (prevalence [MeSH Terms]) AND (Drug Resistance [MeSH Terms] OR Antimicrobial Drug Resistance [MeSH Terms] OR Antibiotic Resistance [MeSH Terms]) AND (*Staphylococcus aureus* [MeSH Terms] OR *S. aureus* [MeSH Terms]), AND (*Methicillin-resistant Staphylococcus aureus* [MeSH Terms] OR MRSA [MeSH Terms]), AND (cystic fibrosis [MeSH Terms] OR CF [Title/Abstract]), AND (Middle East [MeSH Terms]). The bibliographic section of pertinent studies was also hand-searched to recognize further potentially eligible articles. Articles published in languages other than English were not evaluated.

### Inclusion and exclusion criteria

Study inclusion criteria were cross-sectional and cohort studies that presented the prevalence of *S. aures*, antibiotic resistance pattern, MRSA prevalence in CF patients from the Middle East were included. Also, articles that use standardized tests to determine antibiotic susceptibility were included. Studies before 1999, studies other than Middle East countries, studies with missed data, unclear data, abstracts, conferences, case reports, editorials, meetings, and reviews were excluded. As well, studies were written in languages other than English and also published before 1999 were deleted.

### Quality assessment

Selection bias was assessed with the Critical Appraisal Skills Programme (CASP) checklist for cross-sectional studies (www.casp-uk.net). For each study, 10 questions were asked. So, the questions that were answered "yes" were given a score of 1, and if there was “no answer or doubts," the score was 0. According to this scoring system, studies were divided into three categories: poor (1–4), intermediate (6–8), and strong (> 8). In the end, poor studies were eliminated (Additional file 1).

### Data extraction

Two investigators independently reviewed the selected articles and extracted the pertinent data regarding the characteristics of each study. Data were first author, location (country), study’s time, publication time, sample size, age, genus (male, female), and cystic fibrosis prevalence (*n*).

### Statistical data analysis

Meta-analysis was performed using Comprehensive meta-analysis software (Version 3.3.070). The prevalence was calculated by 95% confidence intervals (CIs). Statistical heterogeneity between studies was assessed using Cochran's *Q*-test (*p* < 0.05 was defined to indicate the presence of heterogeneity) and the *I*^2^ (for assessing the degree of heterogeneity). The random-effect model was used because there was significant statistical heterogeneity between the studies. Also, publication bias was checked by Egger's regression asymmetry test and Funnel plot. As well, subgroup analysis was done for MRSA strains and antibiotic resistance patterns.

## Results

### Study selection and characteristics of included studies

**Fig. 1:**
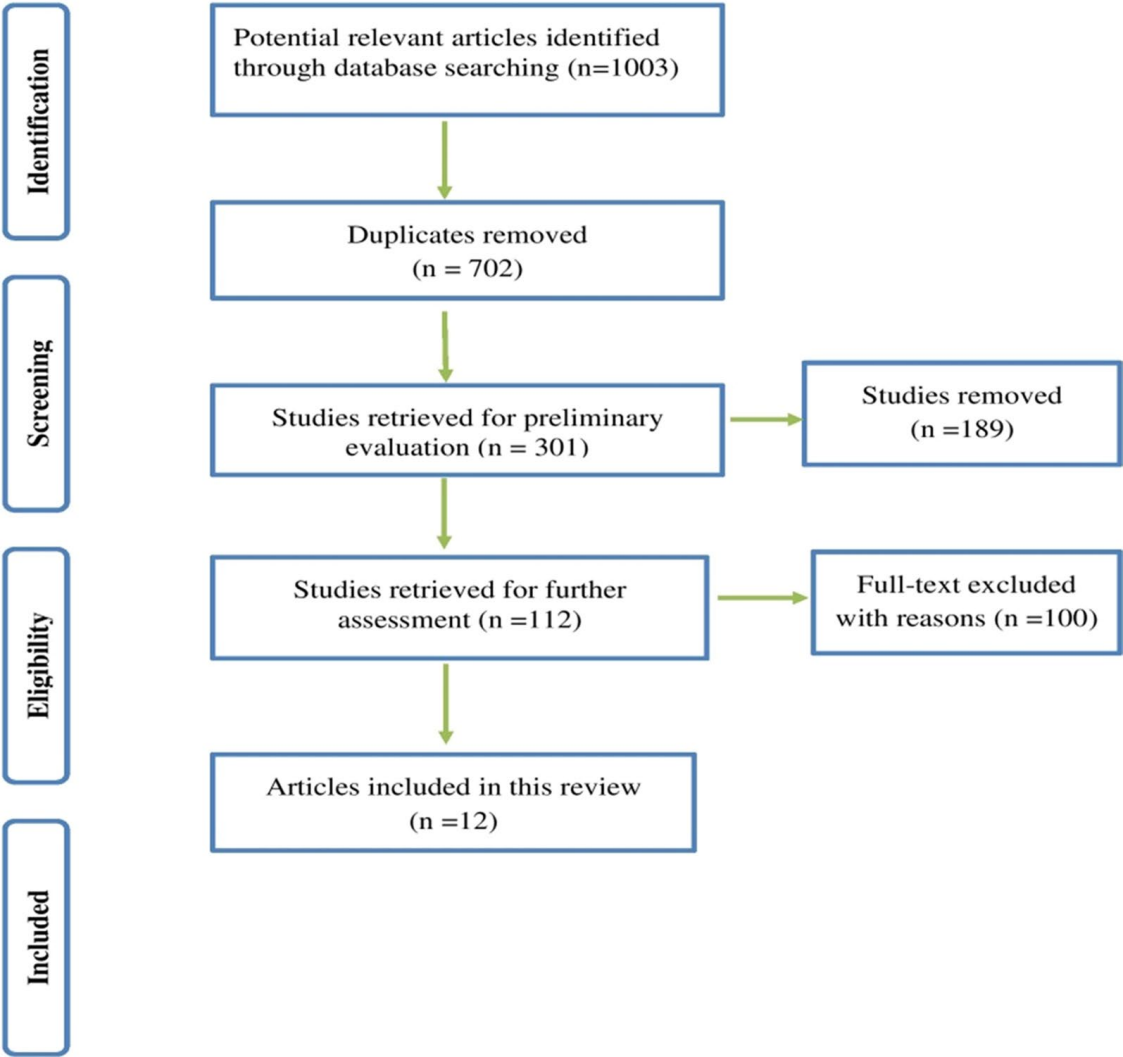
Flowchart of inclusion process for studies

A total of 1003 studies were recognized in the primary search. After assessing titles, abstracts and full-texts, 12 studies met eligibility criteria for inclusion (Fig. 1). The frequency of studies from different countries was as follows; Turkey (*n* = 3), Iran (*n* = 3), Qatar (*n* = 4), Egypt (*n* = 1), and the United Arab Emirates (*n* = 1). Patients’ age ranged from 1.6 to 18 years. Females were more than males. The most samples used were sputum, deep pharyngeal swabs, and Bronchoalveolar lavage (BAL) (Table 1).

**Table 1:** Characteristics of studies included in the current review

First Author	Study time	Publication	Settings	sample size	s. aureus (*n*)	Genus (%)		Age	Samples types	MRSA (*n*)
						Female	Male			
Nobandegani [30]	2011–12	2016	Iran	172	93	60	40	5.9 ± 0.43 years	Sputum samples, an oropharyngeal (OP) swab	74
Khanbabaee [31]	2004–2010	2012	Iran	129	12	60.5	39.5	75.57 (± 6 2.48) months	Sputum samples or pharyngeal swabs	–
Aghamohammadi [32]	2014–2015	2018	Iran	174	28	59	41	7.1 ± 5.7 y	–	–
Wahab [33]	–	2014	Qatar	26	11	54	46	Median 15.5 years	Sputum, deep pharyngeal swabs, BAL	–
Thomas [34]	2010–2017	2019	Qatar	23	15	–	–	–	–	–
Elshafie [35]	2002–2003	2007	Qatar	113	30	60.5	39.5	10 years (1.6–18 years)	Deep oropharyngeal swabs, sputum	–
Wahab [20]	2002–2003	2004	Qatar	36	28	60.5	39.5	10 years (1.6–18 years)	Sputum or oropharyngeal samples	–
Pakasticali [36]	2013–2014	2016	Turkey	84	60	60.7	39.3	11.5 years	Throat specimen, sputum BAL	2
Yurdakul [37]	2003–2010	2012	Turkey	604	325	37.5	62.5	–	Deep throat swabs or sputum samples	24
Yagci [6]	2007–2008	2013	Turkey	248	123	52	48	10.4 years (range: 1–58 years)	Sputum, deep throat swab	–
Frossard [38]	1994–1996	1999	UAE	15	7	40	60	(5.4‹3.5 and 1.0‹1.1 years	–	–
El-Falaki [39]	2010–2012	2014	Egypt	36	2	61	39	3.91 ± 4.18 years	Sputum	–

*UAE* United Arab Emirates

### Overall effects

**Fig. 2:**
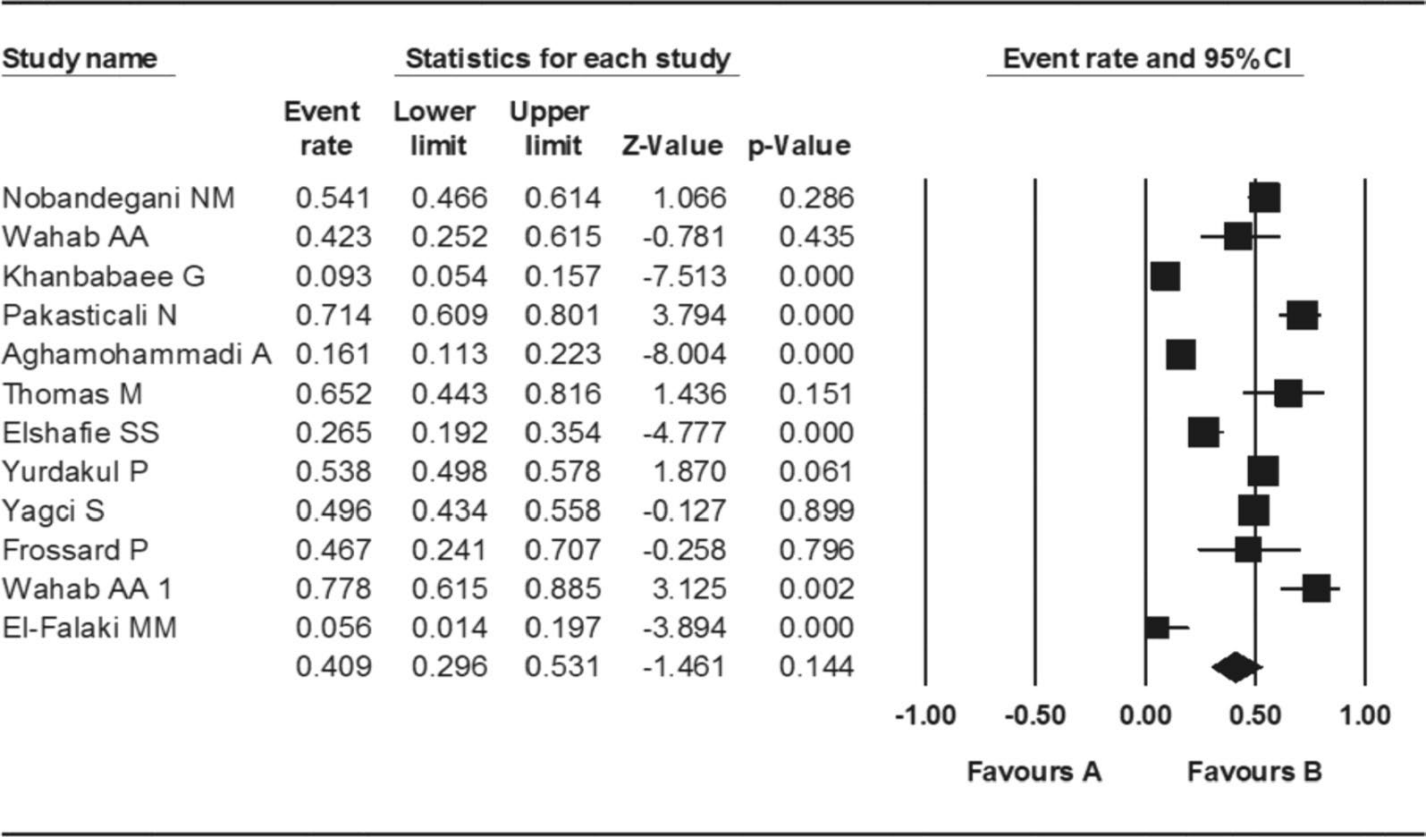
Forest plot of the meta-analysis of prevalence of *S. aureus* isolated from patients suffered from cystic fibrosis

As it is observed in Table 1 and Fig. 2, the prevalence of *S. aureus* was varied between 5.6 and 77.8% in different countries included in this review. In general, the combined prevalence of *S. aureus* in some Middle East countries from 1999 to 2020 was reported by 40.9% (95% CI 29.6–53.1). Findings from selected studies showed that apart from Egypt, there is no significant difference in the prevalence of *S. aureus* in Middle Eastern countries and they are almost in the same range (Fig. 2). Also, comparing the prevalence of *S. aureus* based on the year of study showed that the prevalence of this microorganism hasn’t changed much since the beginning (1999) until now (2020), and its isolation from cystic fibrosis patients has been constant (Table 1).

### Heterogeneity and publication bias

**Fig. 3:**
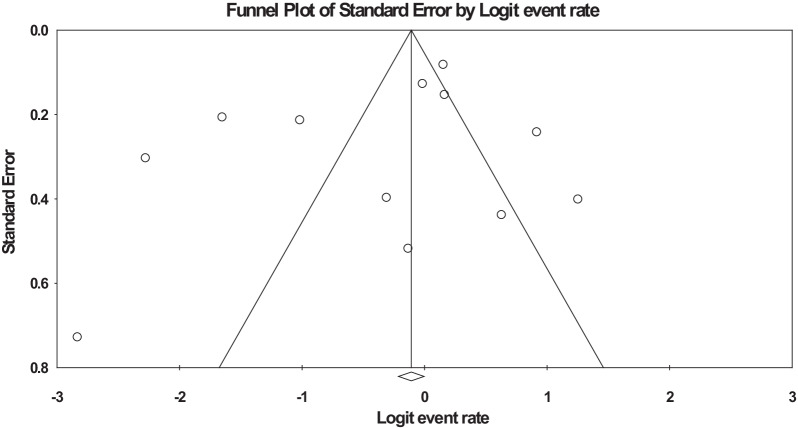
Funnel plot of the meta-analysis of prevalence of *S. aureus* isolated from patients suffered from cystic fibrosis

Regarding data achieved in the present study, heterogeneity was seen (*Q* = 185.9 and *Z* = 1.4, and *I*^2^ = 94). Visual survey of Funnel plot showed the bias in the studies. Egger's linear regression test was performed to further investigate this subject. According to the findings, there was no publication bias among studies due to *p* = 0.31 (Fig. 3).

### Subgroup analysis

#### Prevalence of MRSA

As summarized in Table 2, the pooled prevalence of MRSA strains was reported at 18.6% (95% CI 1.1–82.6), *Z* = 0.9, *I*^2^ = 98.6, *Q* = 146.7.

**Table 2:** Overall effects and subgroup analysis for antibiotic resistance pattern in *S. aureus* recovered from CF patients

Subgroups	Number of studies	Heterogeneity test			Egger’s test		Random model		
		Prevalence (95% CI) (%)	*Z*	*P*	*Q*	*P*	*I*^2^	*T*	*P*
*S. aureus*	12	40.9% (29.6–53.1)	1.4	0.1	185.9	0.00	94	1	0.31
*Subgroup analysis*									
MRSA	3	18.6% (1.1–82.6)	0.9	0.3	146.7	0.00	98.6	0.07	0.95
*Subgroup analysis for antibiotic resistance pattern*									
Amikacin	3	21.5 ( 12.2–38.3)	2.3	0.00	23	0.00	86	1.1	0.34
Cephazolin	3	12.3 (3.1–22.4)	3.1	0.21	44.1	0.00	73	0.1	0.22
Chloramphenicol	3	9.7 (3.3–32.2)	21	0.00	117	0.00	89	2.3	0.11
Ciprofloxacin	4	54.9 (2.5–98.3)	0.1	0.9	33.9	0.00	97	0.12	0.33
Clindamycin	4	17.9 (12.6–24.9)	7.1	0.00	1.2	0.00	19.8	0.12	0.14
Gentamycin	4	10.8 (6.2–18.3)	6.7	0.00	2	0.15	51.6	3.2	0.2
Linezolid	5	0 (0.1–1.2)	1	0.00	2	1	0.00	0.01	0.1
Vancomycin	5	0 (0.1–1.2)	1	0.00	2.1	1	0.00	0.01	0.1
Trimethoprim-Sulfamethoxazole	5	17 (8.6–30.9)	3.9	0.00	5.4	0.06	63.4	5.1	0.12
Penicillin G	4	94 (70.1–99.1)	2.8	0.005	5.7	0.016	82.6	0.1	0.21
Tetracycline	4	22 (8.6–46)	2.2	0.025	6.6	0.01	84.8	1.3	0.01
Teicoplanin	3	0 (0.1–1.2)	1	0.00	2	0.001	0.00	0.01	0.1
Telithromycin	3	0 (0.1–1.2)	1	0.00	2	1	0.00	0.01	0.1
Cefoxitin	3	100 (99.9–100)	1	0.001	3.6	0.001	89.3	0.1	0.32
Clarithromycin	3	0 (0.1–1.2)	1	0.00	2	1	0.00	0.01	0.1
Oxacillin	3	100 (99.9–100)	1	0.001	3.6	0.001	88.2	0.1	0.32
Tigecycline	3	0 (0.1–1.2)	1	0.00	2	1	0.00	0.01	0.1

#### Antibiotic resistance pattern

The pooled prevalence of resistance for each antibiotic was calculated. All isolates were resistant against Cefoxitin and Oxacillin, that’s why they all were MRSA. The highest combined resistance of *S. aureus* strains was reported against Penicillin G, followed by Ciprofloxacin with resistance rates 94% (95% CI 70.1–99.1), and 54.9% (95% CI 2.5–98.3), respectively. All strains displayed susceptibility to Tigecycline, Clarithromycin, Telithromycin, Teicoplanin, Linezolid, and Vancomycin.

## Discussion

Bacteria as the most important pathogens still play a significant role in aggravating lung complications in patients with cystic fibrosis [18]. Many organisms isolated from the sputum of CF patients are normal flora of the nose (*S. aureus*) or opportunistic pathogens such as *P. aeruginosa* which is a common environmental organism [19]. Due to the importance of *S. aureus* especially MRSA isolates in CF patients, in the current review, the prevalence of this microorganism and its antibiotic resistance pattern was investigated. In our study, the prevalence of *S. aureus* in different countries varied between 5.6 and 77.8%. In general, the combined prevalence of *S. aureus* in some Middle East countries from 1999 to 2020 was reported by 40.9%. Also, the pooled prevalence of MRSA strains was reported at 18.6%. So, based on data obtained from the current meta-analysis, *S. aureus* was isolated at a high rate from CF patients. Finding *S. aureus* in the lower respiratory tract certainly indicates a pathological situation that has never been adequately investigated [20, 21]. Our results are in accordance with reports from the USA, where the US Patient Registry Annual Data Report presented the prevalence of *S. aureus* 70.3% in CF patients [22]. Also, accordingly, the report of the prevalence among European countries was varied between 60 and 75% [23–25].

According to the results of included studies, there is no significant difference in the prevalence of *S. aureus* in Middle Eastern countries except Egypt and they all are almost in the same range. Also, comparing the prevalence of *S. aureus* based on the year of study showed that the prevalence of this microorganism has not changed much since the beginning (1999) until now (2020), and its isolation from cystic fibrosis patients has been constant. The stability of prevalence of *S. aureus* is possibly owing to improved awareness and infection prevention and control strategies [22]. If we look at it from another angle, the lack of new molecular techniques and the constant use of phenotypic methods or the lack of proper health policies in this area may have caused the prevalence of these microorganisms in these patients over the years to be almost constant which this requires a change in health policy attitudes. We reported the prevalence rate about of 18.6% of MRSA, similarly, USA Patient Registry Annual Data Report showed a prevalence rate of around 25% for MRSA [22], and Argentina (25.9%) [26]. But in contrast to our study, this rate was lower in Poland (6%) [27] and Germany (4%) [28]. We surely know MRSA strains will bring up more problems for patients with CF [2].

In the present systematic review and meta-analysis, the highest combined resistance in *S. aureus* strains was reported against Penicillin G, followed by Ciprofloxacin with resistance rates of 94%, and 54.9%, respectively. Therefore, according to results achieved, most antibiotics used in included studies except Penicillin G and Ciprofloxacin were effective against *S. aureus* isolates. This must be taken into consideration in line with our results, a study conducted by Cafiso et al. All strains were susceptible to Linezolid and Tigecycline [8]. Regarding the identification of the clinical significance of *S. aureus* pulmonary infection particularly MRSA in CF and the restricted data available to guide present therapeutic regimes [2, 29], in the current review, most antibiotics especially Tigecycline, Clarithromycin, Telithromycin, Teicoplanin, Linezolid, and Vancomycin showed a good impact on *S. aureues* isolates. Therefore, it can use the mentioned antibiotics in MRSA pulmonary infection in CF patients in the Middle East area.

The main limitation of the present study is that we search only studies published in English and other languages such as Persian, Turkish, and Arabic that didn’t include. Also, the sample size used here was small and the number of studies enrolled unfortunately did not cover all Middle Eastern countries.

In summary, our review reported a high prevalence of *S. aureus* and an intermediate prevalence of MRSA strains in pulmonary specimens achieved from CF patients. The prevalence in most of the countries included in this study was almost the same and showed a steady trend within a few years from 1999 to 2020. Due to these facts, it is necessary to use new molecular methods to identify this microorganism.

## Conclusion

Preventive measures and health policies should be implemented in the Middle East region to prevent the spread of infection caused by this microorganism, specifically MRSA in CF patients.

## Supplementary Information

**Additional file 1.** PRISMA 2020 Checklist for reporting systematic reviews.

## Data Availability

All data used and analyzed during this study are based on an original article and is available from the corresponding author, by request.

## Abbreviations

ISI: Web of Science
CF: Cystic fibrosis
CFTR: Cystic fibrosis conductance regulator
MSSA: Methicillin-susceptible staph
CASP: Critical Appraisal Skills Programme
CIs: Confidence intervals
